# Closing in on pacemaker cells

**DOI:** 10.7554/eLife.108102

**Published:** 2025-07-22

**Authors:** Pin-Ji Lei, Timothy P Padera

**Affiliations:** 1 Edwin L Steele Laboratories, Department of Radiation Oncology, Massachusetts General Hospital Cancer Center, Massachusetts General Hospital Boston United States; 2 Harvard Medical School Boston United States

**Keywords:** lymphatic system, muscle cells, pacemaker cells, cell contractions, Mouse

## Abstract

Lymphatic muscle cells orchestrate the contraction of collecting lymphatic vessels in mice.

**Related research article** Zawieja SD, Pea GA, Broyhill SE, Patro A, Bromert KH, Norton CE, Kim HJ, Sivasankaran SK, Li M, Castorena-Gonzalez JA, Drumm BT, Davis MJ. 2025. Cellular characterization of the mouse collecting lymphatic vessels reveals that lymphatic muscle cells are the innate pacemaker cells. *eLife* **12**:RP90679. doi: 10.7554/eLife.90679.

The role of the lymphatic system is to remove extra fluid from tissues and to carry antigens to lymph nodes to generate an immune response when needed. The network of lymphatic vessels and lymph nodes extends from the surface of the brain to the tips of the toes, and also to every organ system in between. Disruption of lymphatic function leads to tissue swelling, the build-up of waste products, and impaired immunity. Despite the importance of the lymphatic system, efforts to therapeutically improve lymphatic function remain limited.

The fluid and the cells inside lymphatic vessels are collectively known as lymph, and although the lymphatic system extends to every part of the body, there is no central pump like the heart to keep lymph moving through the lymphatic system. Instead, the system relies on spontaneous contractions of the larger lymphatic vessels (Figure 1). These vessels contain specialized lymphatic muscle cells that can both regulate the diameter of the vessel and drive the rapid contractions needed to pump lymph.

**Figure 1.**
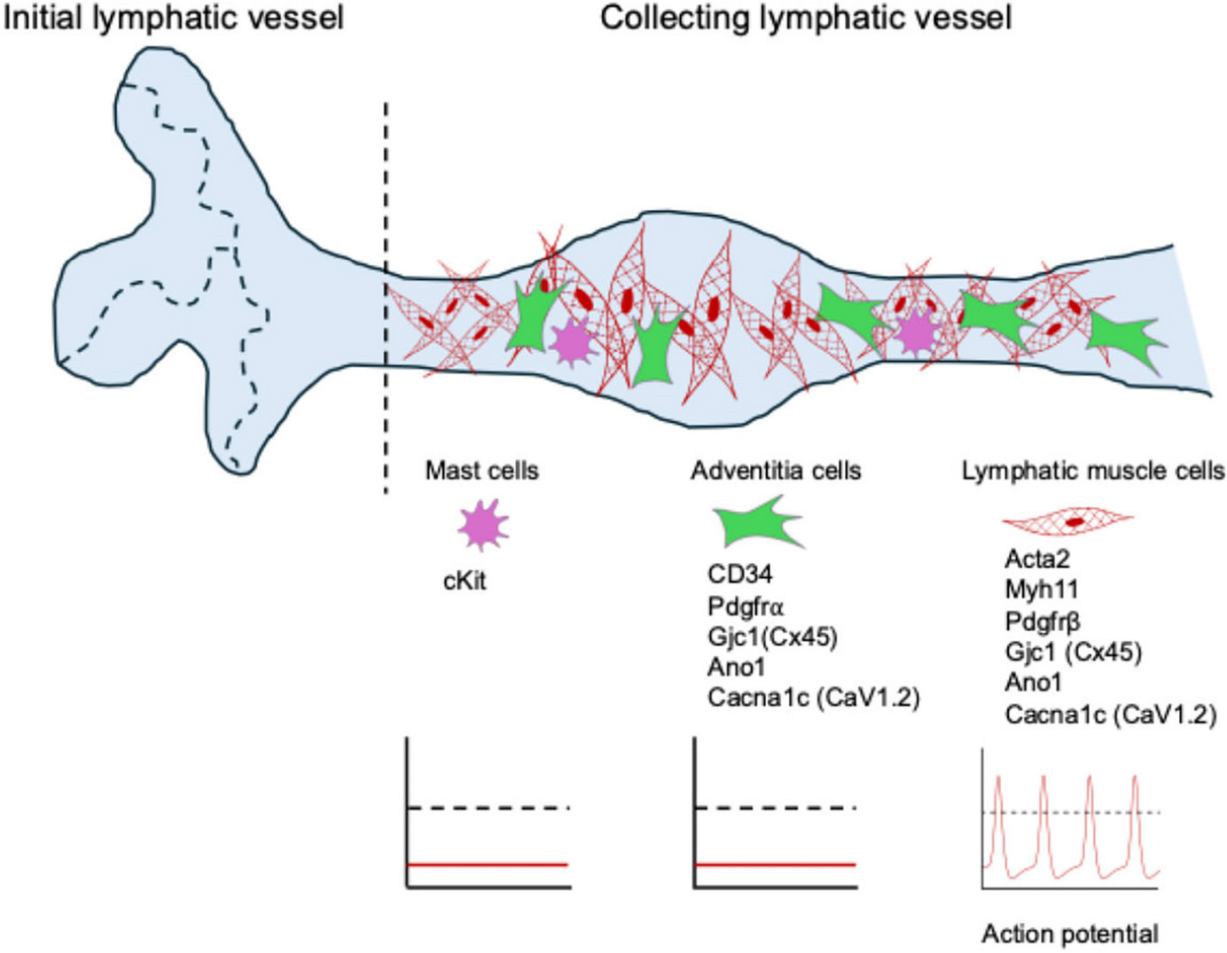
Lymphatic muscle cells are the pacemaker cells in the vessel wall. Initial lymphatic vessels (left of vertical dashed line) consist of a single layer of endothelial cells forming button-like junctions, which allow for the uptake of interstitial fluid and waste products to form lymph (pale blue). Collecting lymphatic vessels (right of vertical dashed line) are composed of endothelial cells with tight junctions and are surrounded by lymphatic muscle cells (red), adventitial cells (green) and mast cells (purple). They also contain valves that prevent the backflow of lymph. Although adventitial cells express genes encoding ion channels and electrical conduction machinery (such as CD34 and the other genes shown), they fail to initiate action potentials (middle graph), as do mast cells (left graph). In contrast, lymphatic muscle cells can produce action potentials (right graph) and can serve as the innate pacemaker cells, responsible for initiating the contraction of collecting lymphatic vessels.

Lymphatic muscle cells have been shown to generate action potentials and propagate them down the vessel. However, the initial trigger for these coordinated action potentials remains unknown. Also, it is not clear if lymphatic vessels contain pacemaker cells like those that initiate smooth muscle contractions in the gastrointestinal tract and upper urinary tract (Briggs Boedtkjer et al., 2013). If collecting lymphatic vessels do contain pacemaker cells, which type of cell is responsible?

Now, in eLife, Scott Zawieja, Michael Davis and colleagues at the University of Missouri and other institutes in the US and Ireland report that lymphatic muscle cells are the pacemaker cells that control the rhythmic contractions of collecting lymphatic vessels in mice (Zawieja et al., 2025). Through a rigorous examination of these vessels, the team identified four major components of the collecting lymphatic vessel wall: mast cells, adventitial cells, lymphatic endothelial cells and lymphatic muscle cells. To better understand the possible roles of these cells in pacemaking, they used a combination of immunofluorescence staining, single-cell RNA sequencing, and gene reporter mice.

Intriguingly, the adventitial cells expressed three genes that, based on previous studies, are known to be involved in regulating membrane potential and electrical conduction in lymphatic muscle cells. However, no significant changes in contractile function were observed in transgenic mice in which these three genes had been deleted. Thus, the adventitial cells seem unlikely to be pacemaker cells.

Next, the team employed an optogenetic approach to test which type of cells could elicit coordinated contractions in lymphatic vessels. They generated mouse strains that expressed a light-gated ion channel called ChR2 in either adventitial cells, mast cells or lymphatic muscle cells. When exposed to blue light, ChR2 opens and allows cations to enter the cell, leading to cell membrane depolarization, potentially triggering action potentials (Nagel et al., 2003). Photo-stimulation of either adventitial cells or mast cells failed to induce coordinated contractions. However, photo-stimulation of lymphatic muscle cells triggered contractions that propagated in a way comparable to the spontaneous contractions.

Calcium-ion signaling has an important role in regulating the activity of pacemaker cells in many organisms, so Zawieja et al. used a calcium-indicator mouse strain to examine calcium-ion signals in the different cell types. They found that the mast cells and adventitial cells did not show consistent calcium transients, whereas the lymphatic muscle cells had synchronous calcium flashes and subcellular calcium oscillations that increased with pressure. Taken together, these results show that lymphatic muscle cells can trigger contractions and are the pacemaker cells in collecting lymphatic vessels.

Identifying the pacemaking ability of lymphatic muscle cells helps explain why lymphatic vessels exhibit both phasic (rapid) contractions and tonic (sustained) contractions. This unique combination also distinguishes them from other types of muscle cells, as demonstrated by other single-cell RNA analyses. Notably, the expression pattern of myosin heavy chain genes in lymphatic muscle cells is similar to that of the vascular smooth muscle cells that control blood vessel tone, while the expression pattern of voltage-gated calcium channels resembles that of the contractile cardiac muscle cells in the heart (Lei et al., 2025). Of note, lymphatic muscle cells are susceptible to aging-related changes in their contractile activity, which can lead to reduced lymphatic drainage (Du et al., 2024; Lei et al., 2025). However, because of their pacemaking ability, reestablishing action potentials in even a small number of lymphatic muscle cells may help combat age-related changes.

The work of Zawieja et al. is one of several recent studies that have used single-cell RNA sequencing to characterize the gene expression profiles in lymphatic muscle cells (Lei et al., 2025; Arroyo-Ataz et al., 2025; Schulz et al., 2025). Integrating these datasets may offer new opportunities to better define the unique transcriptomic profiles of lymphatic muscle cells, characterize their heterogeneity, and provide insights into identifying their progenitor cells for applications in regenerative medicine and drug development.

One caveat of the latest study is that mice are small animals, and their lymphatic vessels only have a single layer of muscle cells. In larger mammals, the lymphatic vessel wall has a more complex structure with multiple layers of muscle cells, so future studies are required to understand the pacemaking ability of lymphatic muscle cells in large animals and humans.
